# The Effect of Obesity Class on the Energetics and Mechanics of Walking

**DOI:** 10.3390/nu13124546

**Published:** 2021-12-18

**Authors:** Julia Primavesi, Aitor Fernández Menéndez, Didier Hans, Lucie Favre, Fabienne Crettaz von Roten, Davide Malatesta

**Affiliations:** 1 Institute of Sport Sciences of the University of Lausanne (ISSUL), University of Lausanne, Bâtiment Synathlon, 1015 Lausanne, Switzerland; julia.primavesi@unil.ch (J.P.); aitorfm86@gmail.com (A.F.M.); Fabienne.crettazvonroten@unil.ch (F.C.v.R.); 2 Center for Bone Diseases, Lausanne University Hospital, 1011 Lausanne, Switzerland; Didier.Hans@chuv.ch; 3 Lausanne University Hospital (CHUV), Consultation de Prévention et Traitement de L’obésité, 1011 Lausanne, Switzerland; lucie.favre@chuv.ch

**Keywords:** energy cost, gait, mechanical work, principal component analysis, recovery

## Abstract

Higher mass-normalized net energy cost of walking (NetC_w/kg_) and mechanical pendular recovery are observed in obese compared to lean adults. This study aimed to investigate the effect of different classes of obesity on the energetics and mechanics of walking and to explore the relationships between body mass, NetC_w/kg_ and gait mechanics by using principal component analysis (PCA). NetC_w/kg_ and gait mechanics were computed in severely obese (SOG; *n* = 18, BMI = 40.1 ± 4.4 kg·m^−2^), moderately obese (MOG; *n* = 17, BMI = 32.2 ± 1.5 kg·m^−2^) and normal-weight (NWG; *n* = 13, BMI = 22.0 ± 1.5 kg·m^−2^) adults during five walking trials (0.56, 0.83, 1.11, 1.39, 1.67 m·s^−1^) on an instrumented treadmill. NetC_w/kg_ was significantly higher in SOG compared to NWG (*p* = 0.019), with no significant difference between SOG and MOG (*p* = 0.14), nor between MOG and NWG (*p* = 0.27). Recovery was significantly higher in SOG than in NWG (*p* = 0.028), with no significant difference between SOG and MOG (*p* = 0.13), nor between MOG and NWG (*p* = 0.35). PCA models explained between 17.0% and 44.2% of the data variance. This study showed that: (1) obesity class influences the gait energetics and mechanics; (2) PCA was able to identify two components, showing that the obesity class is associated with lower walking efficiency and better pendulum-like characteristics.

## 1. Introduction

In recent decades, the prevalence of obesity has continued to increase across the world [1], representing a considerable global public health issue [2]. Obesity is defined as an excessive or abnormal fat accumulation, presenting health risks related to multiple chronic conditions [2]. Excess body weight can be assessed by the body mass index (BMI), defined as the ratio between body mass (kg) and the squared height (m^2^). The World Health Organization (WHO) categorizes obesity and the associated health risks into class I (30.0 ≤ BMI ≤ 34.9 kg·m^−2^), II (35.0 ≤ BMI ≤ 39.9 kg·m^−2^), and III (BMI ≥ 40.0 kg·m^−2^) [2].

Physical activity can be an effective strategy to prevent or minimize weight gain in adults [3] and obesity-related comorbidities [4]. Walking is a common, accessible, and relatively safe type of physical activity [5,6], and is recommended for individuals with obesity to prevent obesity-related comorbidities [4]. However, these individuals walk less compared to their lean counterparts [7], partly due to the greater net energy cost of walking (i.e., the energy expenditure per unit of distance above resting energy expenditure) [8,9,10]. Meeting walking recommendations may therefore be particularly challenging for individuals with obesity. Nevertheless, this lower economy can be improved by training [11]. Understanding how obesity influences the energetics of walking is therefore crucial to prescribe adequate physical activity in weight management programs aimed at improving the health of this population.

Several investigators have found that net energy cost of walking is higher in individuals with obesity compared with their leaner counterparts in absolute (J·m^−1^; NetC_w_) and relative (J·kg^−1^·m^−1^; NetC_w/kg_) values at predetermined walking speeds [8,9,10,12,13]. This lower economy has been attributed to several factors affecting the gait pattern, including increased body mass [9], decreased body stability [14], greater step width [15,16], and wider lateral limb swing [16]. However, the cumulative effect of these factors should have resulted in a higher NetC_w/kg_ (+80%) [9] than that actually assessed (+10–20%) which may be explained by an improved recovery of mechanical energy during walking (Recovery) [12,13]. In analogy to an inverted pendulum, at each step, the forward kinetic energy (E_k_) and the potential energy (E_p_) of the center of mass (COM) are transferred (some E_k_ is converted into E_p_ and vice versa), reducing the external mechanical work performed (the work done by locomotory muscles to lift and accelerate the COM relative to the surroundings; W_ext_) and the associated energy expenditure during walking [17].

Recently, Fernandez-Mendez et al. [13] reported a lower walking economy in individuals with class III obesity compared with their lean counterparts. Furthermore, this class seems to have a better-defined influence on NetC_w/kg_ (+19% averaged across all walking speeds) than class I (+8.5%), because individuals with class I obesity showed only a tendency toward a greater NetC_w/kg_ when compared with their lean counterparts [12]. The higher NetC_w/kg_ in individuals with class III obesity was still lower than theoretically expected (+80%) [9] and obtained with higher pendular recovery (+9.5%) along with a lower relative W_ext_ (W_ext/kg_; J·kg^−1^·m^−1^) at all experimental walking speeds (0.56–1.67 m·s^−1^) [13], whereas individuals with class I obesity mitigated this increase with an improved Recovery (+7%) and lower W_ext/kg_, but only at fast speeds (>1.1 m·s^−1^) [12]. The higher NetC_w_ and Recovery in individuals with class III obesity seems to suggest that the obesity level may be involved in the increased NetC_w/kg_, as well as in pendular exchange optimization. However, it seems that this profitable mechanism runs up to a *functional body mass threshold* beyond which the increase in NetC_w_ cannot be completely—or at least partially—be compensated by pendular transduction [18], as demonstrated by load-carrying studies. Indeed, the Luo and Kikuyu women (i.e., ethnic groups of East Africa), who are daily accustomed to carrying loads, could carry loads up to 20% of their body mass with no apparent increase in NetC_w/kg_ and with a decrease in W_ext/kg_, along with a more skillful Recovery. However, for loads beyond 30% of their body mass, the results showed an increase in NetC_w/kg_ and a tendency to maintain a constant W_ext/kg_, even though Recovery remained improved [18]. This “dissociation” between NetC_w_ and W_ext_/Recovery may be due to (i) a decrease in muscular efficiency, (ii) an increase in the isometric contractions required to support the load and maintain posture, or (iii) the inadequate/inappropriate muscle strength necessary to carry these loads beyond this amount [18]. If we consider obesity as an *added mass walking* [19], these data suggest that, in individuals with different classes of obesity, a threshold may occur beyond which the greater NetC_w_ cannot completely be mitigated by a more skillful pendular recovery. Altogether, these results highlight a relationship between the level of obesity and the energetics and mechanics of walking. However, to the best of our knowledge, thus far, no studies have investigated this relationship.

Moreover, the NetC_w_ has many predictors which are time-dependent and often correlated with each other [20]. For these reasons, data reduction is critical in gait analysis [20], and principal component analysis (PCA) can be particularly useful to reduce data dimensionality with maximally preserving data variance [21]. However, PCA, including anthropometric, energetic, and mechanical walking parameters, has not yet been used in individuals with different classes of obesity.

Therefore, the first aim of the study was to investigate the effect of different obesity levels on the energetics and mechanics of walking in individuals with class II–III (severely obese group; SOG) and class I (moderately obese group; MOG) obesity, and normal-weight adults (normal-weight group; NWG) while walking at different speeds. It was hypothesized that NetC_w/kg_ would be higher in SOG than MOG and NWG and this extra cost would be associated with a lower W_ext/kg_ along with greater Recovery in SOG than NWG and MOG. On the other hand, NetC_w/kg_ would be non-significantly higher in MOG than NWG and it would be associated with a lower W_ext/kg_ along with greater Recovery only at fast walking speeds in the former compared with the latter group.

The second aim was to explore the relationships between body mass, NetC_w_ and gait mechanics at different walking speeds by using PCA in order to identify any specific data patterns.

## 2. Materials and Methods

### 2.1. Participants

Thirty-five sedentary (no participation in any regular exercise or ≤2 h of physical activity per week over the past year) adults with obesity and thirteen normal-weight adults took part in this study. The median BMI of the participants with obesity (median = 35.1) was used to divide them into SOG (BMI = 40.1 ± 4.4 kg·m^−2^; *n* = 18, 3 men and 7 women: class II; 1 man and 7 women: class III) and MOG (BMI = 32.2 ± 1.5 kg·m^−2^; *n* = 17, 2 men and 15 women; class I: *n* = 17), respectively. An NWG (BMI = 22.0 ± 1.5 kg·m^−2^; *n* = 13, 5 men and 8 women) was also included. All subjects were in good health and were free of musculoskeletal disorders or other conditions that could affect the gait pattern. This study used pooled data of two previously published studies of our research group [12,13]. The studies were approved by the local ethics committee (CER-VD 136/14—CER-VD 2016-01715). Informed consent was obtained from all subjects involved in the study.

### 2.2. Experimental Design

Prior to testing, anthropometrical characteristics and body composition were assessed in individuals with obesity through a dual energy X-ray absorptiometric scan (Lunar iDXA; GE Healthcare, Chicago, IL, USA). Subsequently, all study participants visited the laboratory once to perform (1) a 10 min treadmill familiarization session [22] and (2) five 5 min level walking trials at five different and equally spaced speeds (0.56, 0.83, 1.11, 1.39, 1.67 m·s^−1^) on an instrumented single-belt treadmill (T10-FMT-MED, Arsalis, Belgium). The order of the speeds was determined randomly. A minimum of a 5 min resting period between each walking trial was carried out. Metabolic and mechanical data (10 strides) were collected for each walking speed.

### 2.3. Assessment

#### 2.3.1. Anthropometry and Body Composition

Standing height was measured using a Harpenden Stadiometer. Body mass was measured to the nearest 0.1 kg using a precision digital scale with the subject wearing shorts and a T-shirt. Total and regional masses for each body segment (i.e., trunk, upper and lower limbs) as well as body composition (i.e., lean and fat mass) were assessed in obese subjects through a dual-energy X-ray absorptiometric scan.

#### 2.3.2. Energetics

##### Standing Metabolic Rate

Prior to each trial, the metabolic cart (Oxycon Pro, Jaeger, Germany [12]—Quark CPET, CosmedTM^®^, Italy [13]) was calibrated according to the manufacturer’s recommendations (volume and gas calibration). Afterwards, a 5 min breath-by-breath gas exchange [minute ventilation (${\dot{V}}_{E}$), oxygen uptake (${\dot{V}O}_{2}$) and CO_2_ output (${\dot{V}\mathrm{CO}}_{2}$)] was collected in a standing position and the last minute was averaged to determine the standing metabolic rate (SMR) of each participant.

The two metabolic cards used provide gas exchange measurements that are similar, reliable, and valid relative to each other [23].

##### Net Energy Cost of Walking

The gas exchange was measured breath-by-breath during walking trials. Steady states for ${\dot{V}O}_{2}$ and ${\dot{V}\mathrm{CO}}_{2}$ were reached during the last minute of the 5 min walking trial, with a respiratory exchange ratio (RER) lower than 1 for each walking speed. Errant breaths caused by coughing or swallowing were discarded when ${\dot{V}O}_{2}$ values were higher than 3 standard deviations (3SDs) from the local mean. Subsequently, ${\dot{V}O}_{2}$ (mL·min^−1^) data from the last minute of each walking trial and SMR were averaged. The energy equivalent of 1 L of O_2_ was then used to compute the gross metabolic rate (W) [24]. The net metabolic rate was calculated by subtracting SMR from the gross metabolic rate and subsequently divided by the corresponding walking speed to obtain the absolute NetC_w_ (J·m^−1^); the relative was obtained by dividing this latter by the body mass (NetC_w/kg_; J·kg^−1^·m^−1^).

#### 2.3.3. Mechanics

##### Spatiotemporal Parameters

For each walking trial, step length (i.e., the distance between the initial contact of one foot and the initial contact of the opposite one; m) and frequency (number of steps per second; Hz) were assessed during twenty consecutive steps in the last minute of each walking trial using the instrumented treadmill (sampling rate: 1000 Hz).

##### Mechanical Works and Potential Kinetic Energy Transduction

The mechanical energy fluctuations of the COM in the 3 axes were computed from the vertical (F_v_), forward (F_f_) and lateral (F_l_) components of the ground reaction forces (GRFs) for the 20 steps selected. These components, along with the body mass of each subject, were used to calculate the 3D accelerations of the COM following the method described previously by Massaad et al. [25] The beginning and the end of a step were defined as the instant when F_f_ was equal to zero [12]. Consequently, a primary mathematical integration of these accelerations was applied to obtain the velocities of the COM in vertical, forward and lateral directions (i.e., V_v_, V_f_ and V_l_, respectively). A second mathematical integration of V_v_ and V_l_ was applied to assess the COM_v_ and COM_l_. To guarantee step consistency, only steps were selected in which the sum of the increments of the three components did not differ by more than 25% from the sum of decrements [25]. Instantaneous vertical, forward and lateral kinetic energies (i.e., E_kv_, E_kf_, and E_kl_) of the COM were then calculated and used to assess the instantaneous E_k_ of the COM (Equation (1)).

E_k_ = E_kv_ + E_kf_ + E_kl_ = 0.5·*m*·(V^2^_v_ + V^2^_f_ + V^2^_l_) (1)

where *m* is the body mass.

Using the vertical position of COM (*h*), *m* and gravity constant (*g* = 9.81 m·s^−2^), instantaneous E_p_ of the COM was calculated (Equation (2)).

E_p_ = *m·g·h* (2)

Subsequently, the instantaneous total mechanical energy (E_tot_) of the COM was computed as the sum of the instantaneous E_k_ and E_p_ (Equation (3)).

E_tot_ = E_k_ + E_p_ = E_kv_ + E_kf_ + E_kl_ + E_p_ (3)

The amount of the external mechanical work done per step was defined as the sum of the positive increments in E_tot_. Throughout this study, the external mechanical work is expressed in absolute (J·m^−1^; W_ext_) and relative (J·kg^−1^·m^−1^; W_ext/kg_) terms.

Then, the amount of mechanical energy saved via the pendular energy transduction was assessed by Recovery (Equation (4)).

(4) $$\mathrm{Recovery}\;\left(\%\right)=\frac{W_{k}^{}+W_{p}^{}+W_{\mathrm{ext}}^{}}{W_{k}^{}+W_{p}^{}}\;\times \;100$$

where $W_{k}^{}$ and $W_{p}^{}$ represent the sum of the increments in the E_k_ and E_p_ curves, respectively.

The phase shift between the E_k_ and E_p_ curves was computed by determining α (i.e., the time difference between the maximum E_k_ and the minimum E_p_), as previously described by Cavagna et al. [26] (Equation (5)).

α = 360° (t_pk+_·τ^−1^) (5)

where τ represents the step period, t_pk+_ indicates the difference between the period at which E_k_ and E_p_ increase simultaneously (i.e., when the COM starts the upward displacement and concludes the forward acceleration; W_ext_ is performed). Given this definition of α, if E_k_ and E_p_ curves are 180° out of phase, α would be equal to 0° [26]. The difference in amplitude between the above curves was assessed by the W_k_:W_p_^−1^ ratio.

#### 2.3.4. Net Locomotor Efficiency

Net locomotor efficiency (NetE) was computed as the ratio of the W_ext_ to NetC_w_ and expressed as a percentage for each walking speed [12].

### 2.4. Statistical Analysis

Descriptive statistics (mean ± SD) were performed to characterize the sample. Chi-squared tests (precisely, Fisher’s exact test for small numbers) were used for testing sex differences among groups. A one-way ANOVA with Tukey correction was applied to test the difference between groups concerning a first set of the participant’s characteristics (age, height, BMI, total and regional masses, and SMRs). Data normality and homogeneity of variances were assessed by using Shapiro–Wilk’s and Leven’s tests, respectively. If the normality assumption was violated, non-parametric tests were employed. Independent-sample *t*-tests were performed to test the differences in a few experimental variables between SOG and MOG.

Linear mixed models (LMMs) were used to determine differences in energetics and mechanics between groups while walking at different speeds (0.56, 0.83, 1.11, 1.39, and 1.67 m·s^−1^). A random subject effect was introduced in the models (i.e., intercept|Subject) to account for repeated measures for each subject. Fixed effects were added (group and speed with all the interactions of these factors). Holm correction was applied to identify where statistical differences (Group; Speed × Group) occurred. The main effects of speed are omitted in the presentation of results, because it is well accepted that it affects metabolic and mechanics observations [27]. Before these LMM analyses, to exclude an order effect of walking speeds, we included the sequence of the speeds (fixed effect) as an addition to the model. There was no significant order effect for all energetic and mechanical variables.

Theses analyses were performed with Jamovi software, version 1.6.14.0, and R software 4.1.1, with a level of significance set at *p* ≤ 0.05.

Based on sample size (between 5 and 10 subjects for each item) [28] and relevance, six variables were chosen for PCA. PCA was applied to reduce the data dimensionality of the original, correlated variables, while preserving the maximal data variation [21] at five walking speeds; the sixth was discarded because a few participants with severe obesity (*n* = 6) were not able to complete the 5 min walking trial at 1.67 m·s^−1^. The analysis process comprised various steps (calculation of the correlation matrix, extraction and rotation of the initial components, and interpretation of the component’s loadings). Kaiser–Meyer–Olkin (KMO) and Barlett’s tests were performed to confirm the suitability for PCA. Only PCs that explained ≥70% of the data variance were retained [29]. Loadings with a contribution <0.5 and/or that were presented in more than one PC were removed from the interpretation [30]; the others were retained and contributed to the definition of the component name. For each walking speed, the individual value on a given PC (explained variance ≥70%) was computed (PC scores). A series of one-way ANOVAs with Tukey or Games–Howell correction were then performed on these scores. Data normality and homogeneity of variance were tested by using Shapiro–Wilk’s and Levene’s tests, respectively; if assumptions were violated, non-parametric analysis and Welch’s tests were used. This procedure was performed with SPSS software, version 25, with a level of significance set at *p* ≤ 0.05.

## 3. Results

### 3.1. Participant’s Characteristics

The participant’s characteristics are presented in Table 1.

Sex was not significantly different among groups (*p* < 0.22). Age was significantly higher in SOG compared to NWG and MOG (*p* < 0.001 for both), with no significant difference between MOG and NWG (*p* = 0.15). Height was significantly lower in SOG compared to NWG and MOG (*p* < 0.001 for both), with no significant difference between MOG and NWG (*p* = 0.49). BMI and body mass were significantly higher in SOG compared to NWG and MOG (*p* < 0.001 for both) and in MOG compared to NWG (*p* < 0.001). Lean and fat body masses (kg and %) were significantly higher in SOG compared to MOG (*p* = 0.017 and *p* < 0.001, respectively). Head and trunk, upper limb, and lower limb masses were significantly higher in SOG than in MOG (*p* < 0.001, *p* < 0.001 and *p* = 0.037, respectively). Lower limb lean and fat masses (kg and %) were not significantly different between these groups (*p* = 0.30, *p* = 0.16 and *p* = 0.60, respectively).

### 3.2. Energetics

#### 3.2.1. Standing Metabolic Rates

The standing metabolic rates of the participants are presented in Table 1.

The SMR was significantly higher in SOG compared to NWG and MOG (*p* = 0.008 and *p* = 0.006, respectively), with no significant difference between MOG and NWG (*p* = 1.0).

The SMR_/kg_ was significantly lower in SOG compared to NWG (*p* < 0.001) and between MOG and NWG (*p* < 0.001), with no significant difference between SOG and MOG (*p* = 0.83).

#### 3.2.2. Net Energy Cost of Walking

**Figure 1:**
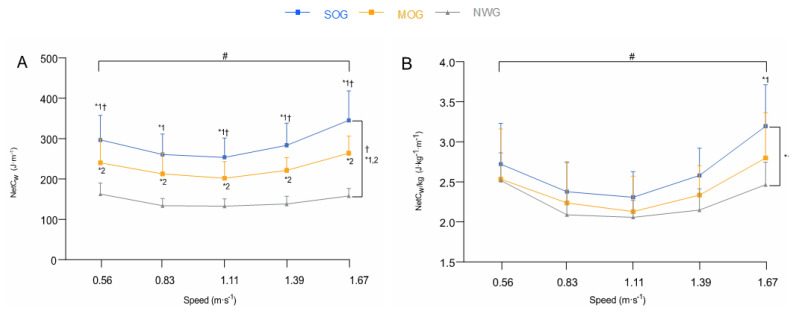
*Absolute net energy cost of walking* (NetC_w_; J·m^−1^) (**A**), *relative net energy cost of walking* (NetC_w/kg_; J·kg·m^−1^) (**B**). Values are the mean ± SD. The blue line corresponds to the severely obese group (SOG; *n* = 18), with orange line to the moderately obese group (MOG; *n* = 17), with grey line to the normal-weight group (NWG; *n* = 13). ^†^ Significant difference between SOG and MOG. *^1^ Significant difference between SOG and NWG. *^2^ Significant difference between MOG and NWG. # Significant speed × group interaction effect.

The NetC_w_ was significantly higher in SOG than in MOG and NWG (*p* < 0.001 for both) and in MOG than NWG (*p* < 0.001). There was a significant interaction effect (*p* < 0.001). The NetC_w_ was significantly higher in SOG compared to MOG at all walking speeds, except at 0.83 m·s^−1^ (*p* = 1.0) (Figure 1A).

The NetC_w/kg_ was significantly higher in SOG compared to NWG (*p* = 0.019), with no significant difference between the former and MOG (*p* = 0.14), nor between MOG and NWG (*p* = 0.27). There was a significant interaction effect (*p* = 0.006). The NetC_w/kg_ was significantly higher in SOG than in NWG at the fastest speed (1.67 m·s^−1^) (*p* = 0.019) (Figure 1B).

### 3.3. Mechanics

#### 3.3.1. Spatiotemporal Parameters and Vertical and Lateral Displacements of the Center of Mass

The spatiotemporal parameters, COM_v_ and COM_l_ at the experimental walking speeds are presented in Table 2.

Step length was significantly lower in SOG compared to MOG (*p* = 0.002), with no significant difference between the former and NWG (*p* = 0.06), nor between MOG and NWG (*p* = 0.28). When normalized by height, step length was not significantly different among the three groups (*p* = 0.61).

Step frequency was significantly higher in SOG compared to NWG and MOG (*p* = 0.026 and *p* = 0.014, respectively), with no significant difference between MOG and NWG (*p* = 0.85).

There was no significant group main effect on COM_v_ (*p* = 1.0).

COM_l_ was significantly higher in SOG compared to NWG (*p* = 0.052), with no significant difference in the former compared with MOG (*p* = 0.30) and in MOG compared with NWG (*p* = 0.30).

For all these analyses, no significant interaction was found (*p* ≥ 0.07).

#### 3.3.2. Mechanical Works

**Figure 2:**
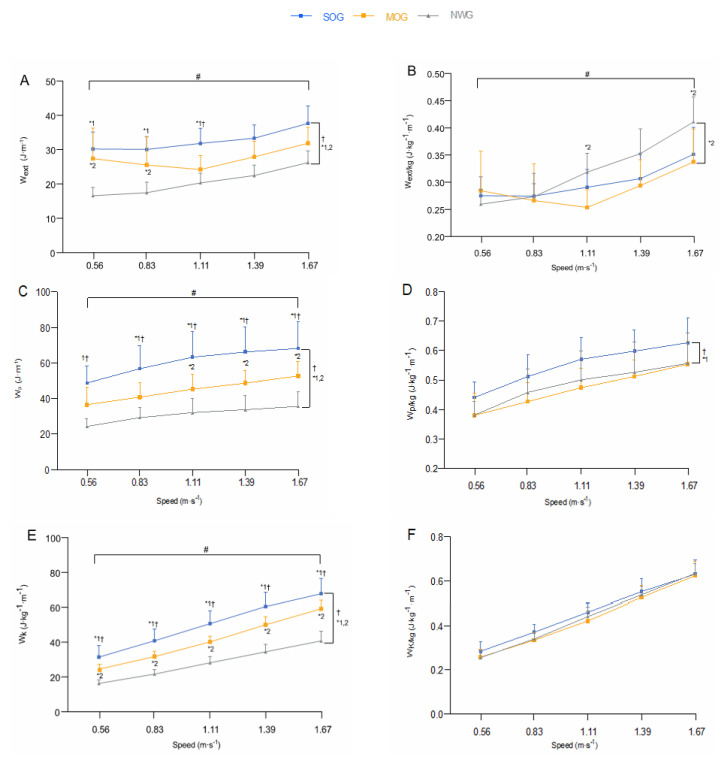
Absolute external mechanical work (W_ext_; J·m^−1^) (**A**), relative external mechanical work (W_ext/kg_; J·kg^−1^·m^−1^) (**B**), absolute mechanical potential energy (W_p_; J·m^−1^) (**C**), relative mechanical potential energy (W_p/kg_; J·kg^−1^·m^−1^) (**D**), absolute mechanical kinetic energy (W_k_; J·m^−1^) (**E**), relative mechanical kinetic energy (W_k/kg_; J·kg^−1^·m^−1^) (**F**) as a function of the walking speed. Values are the mean ± SD. The blue line corresponds to the severely obese group (SOG; *n* = 18), orange line to the moderately obese group (MOG; *n* = 17), and grey line to the normal-weight group (NWG; *n* = 13). ^†^ Significant difference between SOG and MOG. *^1^ Significant difference between SOG and NWG. *^2^ Significant difference between MOG and NWG. # Significant speed × group interaction effect.

The W_ext_, W_p_ and W_k_ were significantly higher in SOG than NWG and MOG (*p* < 0.001 and *p* ≤ 0.01, respectively) and in MOG than NWG (*p* < 0.001 for all). There was a significant interaction effect for these three variables (*p* ≤ 0.005) (Figure 2A,C,E).

The W_ext/kg_ was significantly lower in MOG than in NWG (*p* = 0.022), with no significant difference between SOG and NWG (*p* = 0.16), nor between the former and MOG (*p* = 0.26). There was a significant interaction effect (*p* < 0.001). W_ext/kg_ was significantly lower in MOG than in NWG at 1.11 and at 1.67 m·s^−1^ (*p* = 0.003) (Figure 2B).

The W_p/kg_ was significantly higher in SOG compared to NWG and MOG (*p* = 0.018 and *p* = 0.002, respectively) with no significant difference between MOG and NWG (*p* = 0.53). There was no significant interaction effect (*p* = 0.73) (Figure 2D).

There was neither a significant group main effect on W_k/kg_ (*p* = 0.13) nor a significant interaction effect (*p* = 0.22) (Figure 2F).

#### 3.3.3. Potential Kinetic Energy Transduction and Factors affecting Recovery

**Figure 3:**
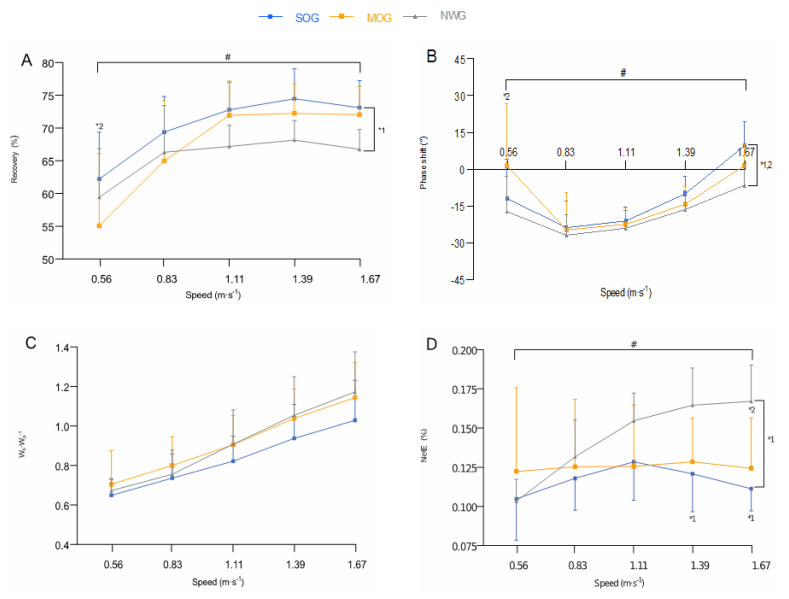
*Recovery (%)* (**A**), *phase shift (°)* (**B**), *amplitude (W_k_·W_p_^−1^)* (**C**), *net locomotor efficiency (NetE; %)* (**D**) *as a function of the walking speed.* Values are mean ± SD. The blue line corresponds to the severely obese group (SOG; *n* = 18), orange line to the moderately obese group (MOG; *n* = 17), and solid grey line to the normal-weight group (NWG; *n* = 13). *^1^ Significant difference between SOG and NWG. *^2^ Significant difference between MOG and NWG. # Significant speed × group interaction effect.

Recovery was significantly higher in SOG than in NWG (*p* = 0.028), with no significant difference between the former and MOG (*p* = 0.13), nor between the latter and NWG (*p* = 0.35). There was a significant interaction effect (*p* < 0.001). Recovery was significantly lower in MOG than in NWG at the slowest speed (0.56 m·s^−1^) (*p* = 0.050) (Figure 3A).

The phase shift was significantly higher in SOG than in NWG (*p* = 0.039) and in MOG than in NWG (*p* = 0.039) with no significant difference between SOG and MOG (*p* = 0.94). There was a significant interaction effect (*p* = 0.002). The phase shift was significantly higher in MOG than NWG at the slowest speed (0.56 m·s^−1^) (*p* = 0.002) (Figure 3B).

There was neither a significant group main effect on W_k_·W_p_^−1^ (*p* = 0.14), nor a significant interaction effect (*p* = 0.23) (Figure 3C).

### 3.4. Net Locomotor Efficiency

The NetE was significantly lower in SOG than in NWG (*p* = 0.014), with no significant difference between the former and MOG (*p* = 0.34), nor between the latter and NWG (*p* = 0.09). There was a significant interaction effect (*p* < 0.001). The NetE was significantly lower in SOG than in NWG at the fastest speeds (1.39 and 1.67 m·s^−1^, respectively) (*p* < 0.001), and in MOG than in NWG only at 1.67 m·s^−1^ (*p* = 0.017) (Figure 3D).

### 3.5. Patterns among Body Mass, Net Cost of Walking, and Gait Mechanics

KMO measures were 0.5, 0.5, 0.7, and 0.6 at 0.56, 0.83, 1.11, and 1.39 m·s^1^, respectively, indicating an acceptable sample adequacy for PCA. Barlett’s test of sphericity was statistically significant for each walking speed (*p* < 0.001). PCA models explained between 17.0% and 44.2% of the data variance. Body mass, W_ext_, and NetC_w_ were loaded highest on PC1 (labeled walking efficiency) at all walking speeds, whereas Recovery and COM_l_ were loaded highest on PC2 (labeled pendulum-like characteristics) at 0.83, 1.11, and 1.39 m·s^−1^ (Table 3).

### 3.6. Usefulness of PC Scores to Identify Gait Pattern Similarities between Groups

At 0.56 m·s^−1^, PC1 scores were significantly higher in SOG than in NWG and MOG (*p* < 0.001 and *p* = 0.012, respectively) and in MOG than in NWG (*p* < 0.001). PC2 and PC3 scores were not significantly different between groups (*p* = 0.11 and *p* = 0.82, respectively) (Table 4).

At 0.83 m·s^−1^, PC1 scores were significantly higher in SOG than in NWG and MOG (*p* < 0.001 and *p* = 0.010, respectively) and in MOG than in NWG (*p* = 0.001). PC2 and PC3 scores were not significantly different between groups (*p* = 0.26 and *p* = 0.16, respectively) (Table 4).

At 1.11 m·s^−1^, PC1 scores were significantly higher in SOG compared to NWG and MOG (*p* < 0.001 for both) and in MOG than in NWG (*p* < 0.001). PC2 scores were also significantly higher in SOG than in NWG (*p* = 0.016) and in MOG than in NWG (*p* = 0.019), with no significant difference between SOG and MOG (*p* = 0.99). PC3 scores were not significantly different between groups (*p* = 0.13) (Table 4).

At 1.39 m·s^−1^, PC1 scores were significantly higher in SOG than in NWG (*p* < 0.001) and MOG (*p* = 0.026) and in MOG than in NWG (*p* = 0.001). PC2 scores were significantly higher in SOG compared to NWG (*p* = 0.024) with no significant different between the former and MOG (*p* = 0.59) nor between MOG and NWG (*p* = 0.18) (Table 4).

## 4. Discussion

The main findings of the present study were that individuals with severe obesity exhibited both a greater NetC_w/kg_ and higher Recovery than normal-weight adults, regardless of the lower W_ext/kg_ performed which, in turn, was significantly lower in subject with moderate obesity than in their normal-weight counterparts, despite similar Recovery and NetC_w/kg_. PCA was able to identify two components: walking efficiency and pendulum-like characteristics. PC scores were sensitive to distinguish individuals with obesity with lower walking efficiency from their normal-weight counterparts and to differentiate adults with severe obesity with better pendulum-like characteristics from other groups, especially at the fastest walking speeds.

As expected, NetC_w/kg_ in SOG was 15% greater (Figure 1B) than NWG and it was non-significantly 6% greater (averaged values across all walking speeds; Figure 1B) in MOG than in NWG, which corroborates previous studies that reported similar results, but showed a greater difference between obese and lean adults [8,9,12,13]. The lower increase in NetC_w/kg_ observed could be attributed to the lower level of obesity of our participants [9]. These findings revealed that (i) the extra cost of walking in individuals with obesity is not only related to body mass increase, and (ii) their lower economy may also be explained by several mechanical gait factors [9,14,15,16,31]. The relative contribution of these factors to NetC_w/kg_ seems to be dependent on the differences in the level of obesity between groups. However, even though our analysis showed that the BMI differences between each comparison were significant (Table 1), these may not be substantial enough to alter the gait mechanics [31] and walking economy in SOG and MOG (Figure 2B).

Although SOG had a greater NetC_w/kg_ compared to NWG, the energetic cost of walking was always lower than theoretically expected (+80%) [9]. This was due to a more skillful Recovery, which was significantly higher in SOG than in NWG (+7%; averaged across all speeds; Figure 3A) and was associated with the optimal phase shift (i.e., α ∼ 0; Figure 3B). These findings are in line with recent data from Fernandez-Mendez et al. [13], who reported similarly higher Recovery (+9.5%; averaged across all speeds) in adults with class III obesity compared to their lean counterparts across all walking speeds tested. This more skillful pendular mechanism may be due to an ingenious utilization of lateral motion which can improve mechanical energy recovery—a useful strategy that has been observed in penguins [32]. Our findings seem to corroborate this hypothetical strategy, which was further supported by our PCA results. In fact, SOG showed significantly larger COM_l_ (+31%; averaged across all speeds) than in NWG across all walking speeds tested (Table 2), along with significantly higher PC2 score at 1.11 and 1.39 m·s^−1^ compared to NWG (Table 4). These data highlight strong relationships between Recovery and COM_l_ (Table 3) in individuals with class II and III obesity (SOG) at these “intermediate speeds” when Recovery reaches the greater/optimum values [33]. The larger lateral movements in SOG may be related to greater thigh girth due to an excessive amount of adipose tissue and/or poor dynamic stability during walking [14,16], and may explain the nonsignificant difference in Recovery between SOG and MOG (Figure 3A). Indeed, our results did not reveal any significant difference between the lower limb fat mass of these groups (Table 1). Unexpectedly, Recovery did not increase at fastest speeds in MOG compared with NWG, which supports the idea that COM_l_ plays a key role in the conservation of mechanical energy during walking. Indeed, MOG compared with NWG presented a nonsignificant COM_l_ (Table 2), along with a nonsignificant PC2 score across all walking speeds tested, except at 1.11 m·s^−1^ (Table 4). Altogether, it seems that the lower obesity level of moderately obese participants (who are placed midway between severely and normal-weight subjects) was not substantial enough to significantly affect the walking speed–Recovery relationship. This corroborates similar findings reported by Fernandez-Mendez et al. [12], reinforcing the hypothesis that the level of obesity plays a key role in pendular exchange optimization.

In contrast to our hypothesis, W_ext/kg_ was not significantly different between SOG and NWG (Figure 2B), despite a more skillful pendular energy transduction (Figure 3A). This unpredictable nonsignificant difference on W_ext_, along with the apparent discrepancy between Recovery and W_ext/kg_, might be explained by the relative amplitude between the E_k_ and E_p_ curves. Although nonsignificant in itself, the W_k_·W_p_^−1^ ratio was 10% greater in SOG than in NWG (Figure 3C). This is further supported by a significantly higher W_p/kg_ in the former (Figure 2D), thus suggesting that obesity may positively affect the pendular mechanism by making these energies more out of phase (Figure 3B), but at the expense of a poorer magnitude optimization (i.e., W_k_·W_p_^−1^ < 1; Figure 3C). The higher W_p/kg_ in SOG compared to NWG would appear to be a consequence of greater knee flexion during the foot strike [34]—a load-protective mechanism used to absorb the impact-related ground reaction forces [35]. This more bouncy walking seems to be related to the larger body mass, because after very large weight loss, the knee flexion in the early stance phase was reduced along with a lower W_p/kg_ [34]. Consequently, greater work against gravity needs to be done by the muscles of the trailing leg to raise COM during the single contact phase. However, less work is needed to accelerate the COM during double contact due to a greater amount of E_p_ available to be converted into E_k_. This *energy mismatch* [36] may increase the W_ext/kg_ performed to redirect the COM velocity from one arc to the next [37], and contribute to explaining the nonsignificant lower W_ext/kg_ observed in adults with class II and III obesity (SOG) compared to their normal-weight counterparts (Figure 2B). On the other hand, as hypothesized, the W_ext/kg_ was significantly lower in MOG compared to NWG, especially at the fastest speeds (Figure 2B), which confirms the previous results [12]. This lower W_ext/kg_ appears to be related to improved timing between the heel strike and the toe-off impulse generated by the plantar flexor muscles of the trailing leg. This allows reduction of the dissipation of mechanical energy and, consequently, the work performed to redirect the COM during step-to-step transitions [37,38], thus limiting the increase in NetC_w/kg_. Interestingly, an artificial reduction in the ankle push-off substantially decreases the walking economy [39], which might explain why individuals with severe obesity have a less efficient walking propulsion and exert more energy over a stride compared to their lean counterparts. This finding further supports the concept of a *functional body mass threshold*, beyond which the NetC_w/kg_ cannot be compensated completely by the improved pendular energy transduction. As speculated by Heglund et al. [18] and confirmed by our study, the greater NetC_w/kg_, concomitantly with the nonsignificant lower W_ext/kg_ found in SOG compared to NWG, resulted in a significantly lower NetE (−19%; across all walking speeds) in the former (Figure 3D), thus corroborating previous studies that have observed lower mechanical efficiency in individuals with obesity than in their lean counterparts [10,12]. PCA appears to confirm the lower NetE with significantly higher PC1 score in SOG and MOG than NWG at all walking speeds (Table 4). However, despite this lower mechanical efficiency in individuals with obesity compared to lean individuals, it may be premature to conclude that mechanical efficiency is impaired in the former, given that the mechanical work has been only partially quantified (W_int_ was not assessed in our study). However, previous results from Fernandez-Mendez et al. [13] have shown that adults with class III obesity present a similar amount of relative W_int_ and greater NetC_w/kg_ compared to their lean counterparts, which suggests that swinging of the limbs is not responsible for the extra energy cost in this population. For this reason, we can assume that the NetE (W_ext_·NetC_w_^−1^) assessed in our study can well approximate the overall mechanical efficiency (W_tot_·NetC_w_^−1^). The lower NetE found in SOG compared to NWG may be explained by a more knee-flexed lower limb at the heel strike [34], which may produce disadvantageous joint loads that would require more muscular tension to prevent the joints from collapsing and to support the body mass [36,40]. Interestingly, an unfavorable joint moment due to considerable load carrying was directly linked to a proportional increase in NetC_w_ [41]. Thus, it seems plausible that individuals with class II and III obesity (SOG) exert more energy due to an increase in muscle co-contractions when their body mass extends beyond a *functional body mass threshold*. Indeed, below this threshold, adults with class I obesity (MOG) presented a lower increase in NetC_w/kg_, which might be better (albeit not completely) cushioned, by a lower W_ext/kg_. Moreover, the lower the level of obesity, the higher the critical speed at which mechanical efficiency is penalized (Figure 3D), because a lower level of obesity would enable generation of an advantageous burst of positive power [42] and to produce appropriate forces to support the body’s weight during walking.

*Practical applications.* Health behavior such as physical activity and diet are essential for body weight control [43]. A greater NetC_w/kg_ therefore represents a potential target for morbid obesity management through the daily total energy expenditure increase (TEE), especially in sedentary people with obesity who are in the so-called “unregulated zone”, in which appetite and food intake are not affected by TEE; thus, food intake drives body weight gain [44]. Although increasing physical activity using walking and its associated higher NetC_w/kg_ could be useful to shift individuals with obesity into the “regulated zone”, in which appetite and food intake are affected by physical activity [44], the role of the latter in energy balance and weight loss remains controversial [44,45,46]. Nevertheless, decreasing the greater NetC_w/kg_ in adults with obesity may be beneficial to increase non-exercise activity thermogenesis (NEAT) [11], and thus, light physical activity energy expenditure (1.6–2.9 metabolic equivalents; METs), which is inversely related to sedentary time [47] and has complementary effects to improve health [48].

Some methodological limitations need to be addressed. First, our groups were not matched for age, sex, or height. However, all subjects in our study were no older than ∼65 years, which is considered the critical age when the energetics and mechanics of walking change substantially [49,50]. Sex distribution in the three groups was not significantly different. The difference in height among the three groups may be involved in the differences found in step length. However, the height difference among the groups was small and negligible and did not influence COM_v_ and mechanical work during walking [51]. This corroborates that age, sex, and height did not represent confounding factors of gait analysis in comparing our three groups. Second, the combined limbs method (CLM) used neglects the simultaneous positive and negative work done by the trailing and leading legs during double support stances, and therefore may have underestimated the measured W_ext_ [52]. However, CLM is the basis for computing Recovery [53]. Third, our study was based on a relatively small sample size that reflected the recruitment challenges—a difficulty that has also been reported by others [54]. Therefore, our findings should be interpreted with caution and require further exploration in more representative samples.

## 5. Conclusions

In conclusion, this study shows that obesity class appears to have an effect on the energetic and mechanics of walking. Individuals with obesity (class II-III, and I) have a greater NetC_w/kg_ compared to their normal-weight counterparts. This extra cost is partially mitigated only in adults with class II–III obesity (SOG) by a more skillful pendular mechanism, consequent to the optimal exploitation of lateral movements that enhance the phase shift between the E_p_ and E_k_ curves at the expense of amplitude optimization. This energy mismatch seems to explain the nonsignificant lower W_ext/kg_ that may relate to the attainment and/or the exceeding of a functional body mass threshold. Indeed, obesity level may be paradoxically associated with lower mechanical efficiency and with better pendulum-like characteristics during walking.

## Figures and Tables

**Table 1:** Participant’s characteristics across groups.

	SOG *n* = 18 (4 M, 14 W)	MOG *n* = 17 (2 M, 15 W)	NWG *n* = 13 (5 M, 8 W)
Variables			
Age, years	37.2 ± 7.8 ^†^ *^l^	32.4 ± 7.5	29.5 ± 5.7
Height, m	1.66 ± 0.07 ^†^ *^l^	1.72 ± 0.07	1.70 ± 0.08
BMI, kg·m^−2^	40.1 ± 4.4 ^†^ *^l^	32.2 ± 1.5 *^2^	22.0 ± 1.5
Body mass, kg	109.7 ± 12.74 ^†^ *^l^	95.28 ± 8.23 *^2^	64.21 ± 8.35
Lean body mass, kg	53.8 ± 7.6 ^†^	50.6 ± 7.7	–
Fat body mass, kg	53.1 ± 11.5 ^†^	41.8 ± 4.6	–
Fat body mass, %	49.3 ± 6.4 ^†^	45.4 ± 5.0	–
Head and trunk mass, kg	54.2 ± 6.7 ^†^	44.1 ± 5.2	37.1 ± 4.8
Upper limbs mass, kg	12.2 ± 1.6 ^†^	10.4 ± 1.4	3.21 ± 0.4
Lower limbs mass, kg	38.4 ± 6.4 ^†^	36.0 ± 4.2	10.3 ± 1.3
Lower limbs lean mass, kg	19.1 ± 3.3	18.9 ± 3.0	–
Lower limbs fat mass, kg	17.3 ± 5.8	16.0 ± 3.4	–
Lower limbs fat mass, %	45.2 ± 9.0	45.8 ± 7.6	–
SMR, W	132.5 ± 18.23 ^†^ *^l^	112.85 ± 19.7	112.32 ± 12.53
SMR_/kg_, W·kg^−1^ body mass	1.21 ± 0.13 *^1^	1.18 ± 0.15 *^2^	1.76 ± 0.15

Values are mean ± SD. ^†^ Significant difference between SOG and MOG. *^1^ Significant difference between SOG and NWG. *^2^ Significant difference between MOG and NWG.—not made for NWG. BMI, body mass index; M, men; MOG, moderately obese group (class I: *n* = 17); NWG, normal-weight group (*n* = 12); SMR, absolute standing metabolic rate; SMR_/kg_, relative standing metabolic rate; SOG, severely obese group (class III: *n* = 8; class II: *n* = 10); W, women.

**Table 2:** Spatiotemporal parameters and vertical and lateral displacements of the center of mass at the experimental walking speeds.

Variables	Step Length, m ^†^	Step Length/Height	Step Frequency, Hz ^†^ *^1^	COM_v_, cm	COM_l_, cm *^1^
0.56 m·s^−1^					
SOG	0.43 ± 0.04	0.26 ± 0.03	1.29 ± 0.13	2.06 ± 0.30	7.61 ± 2.55
MOG	0.47 ± 0.05	0.28± 0.04	1.20 ± 0.14	1.88 ± 0.36	6.61 ± 1.75
NWG	0.47 ± 0.02	0.28 ± 0.02	1.17 ± 0.14	1.98 ± 0.29	6.09 ± 1.20
0.83 m·s^−1^					
SOG	0.54 ± 0.03	0.33 ± 0.02	1.54 ± 0.10	2.95 ± 0.42	5.50 ± 1.66
MOG	0.57 ± 0.03	0.33 ± 0.03	1.47 ± 0.09	2.57 ± 0.39	4.79 ± 1.27
NWG	0.56 ± 0.02	0.33 ± 0.01	1.48 ± 0.06	2.81 ± 0.52	4.23 ± 0.81
1.11 m·s^−1^					
SOG	0.63 ± 0.03	0.38 ± 0.02	1.76 ± 0.08	3.80 ± 0.51	4.26 ± 1.32
MOG	0.66 ± 0.03	0.38 ± 0.02	1.70 ± 0.08	3.33 ± 0.43	3.73 ± 0.89
NWG	0.64 ± 0.02	0.38 ± 0.01	1.71 ± 0.06	3.52 ± 0.69	3.10 ± 0.68
1.39 m·s^−1^					
SOG	0.71 ±.0.03	0.43 ± 0.02	1.94 ± 0.09	4.47 ± 0.62	3.30 ± 0.79
MOG	0.75 ± 0.03	0.44 ± 0.02	1.86 ± 0.08	4.08 ± 0.45	2.95 ± 0.51
NWG	0.73 ± 0.03	0.43 ± 0.01	1.88 ± 0.07	4.14 ± 0.86	2.66 ± 0.71
1.67 m·s^−1^					
SOG	0.79 ± 0.04	0.48 ± 0.02	2.09 ± 0.10	5.29 ± 0.84	2.84 ± 0.72
MOG	0.83 ± 0.03	0.48 ± 0.02	2.02 ± 0.08	4.88 ± 0.69	2.51 ± 0.45
NWG	0.81 ± 0.03	0.48 ± 0.01	2.04 ± 0.08	4.84 ± 0.98	2.34 ± 0.53

Values are mean ± SD. ^†^ Significant difference between SOG and MOG. *^1^ Significant difference between SOG and NWG. *^2^ Significant difference between MOG and NWG. COM_l_, lateral displacements of the center of mass; COM_v_, vertical displacements of the center of mass; MOG, moderately obese group (class I: *n* = 17); NWG, normal-weight group (*n* = 13); SOG, severely obese group (class III: *n* = 8; class II: *n* = 10).

**Table 3:** Explained variance and loadings above the cutoff of different variables on each PC at 0.56, 0.83, 1.11, and 1.39 m·s^−1^.

		PC1	PC2	PC3
	**0.56 m·s^−1^**			
	Explained variance, %	44.2	21.5	19.3
	Variables			
	Body mass	0.96		
Walking efficiency	W_ext_	0.87		
	NetC_w_	0.80		
Pendulum-like characteristics	Recovery		0.98	
	COM_l_			0.75
	Step frequency			−0.74
	**0.83 m·s^−1^**			
	Explained variance, %	44.1	22.7	17.7
	Variables			
Walking efficiency	Body mass	0.93		
	W_ext_	0.92		
	NetC_w_	0.84		
Pendulum-like characteristics	Recovery		0.92	
	COM_l_		0.59	
	Step frequency			0.92
	**1.11 m·s^−1^**			
	Explained variance, %	40.7	29.8	17.0
	Variables			
Walking efficiency	W_ext_	0.95		
	Body mass	0.89		
	NetC_w_	0.82		
Pendulum-like characteristics	Recovery		0.87	
	COM_l_		0.83	
	Step frequency			0.99
	**1.39 m·s^−1^**			
	Explained variance, %	37.2	33.0	–
	Variables			–
Walking efficiency	W_ext_	0.98		
	Body mass	0.79		
	NetC_w_	0.77		
Pendulum-like characteristics	Recovery		0.92	
	COM_l_		0.76	
	Step frequency			

COM_l_, lateral displacements of the center of mass; NetC_w_, absolute net energy cost of walking; PC, principal component (1, 2, and 3, respectively); W_ext_, absolute external mechanical work.

**Table 4:** Comparison of PC scores between groups at 0.56, 0.83, 1.11, and 1.39 m·s^−1^.

	SOG	MOG	NWG
0.56 m·s^−1^			
PC1	0.85 ± 0.58 ^†^ *^1^	0.08 ± 0.58 *^2^	−1.28 ± 0.36
PC2	0.37 ± 0.76	−0.45 ± 1.26	0.08 ± 0.70
PC3	−0.13 ± 1.36	0.07 ± 0.87	0.09 ± 0.50
0.83 m·s^−1^			
PC1	0.81 ± 0.55 ^†^ *^1^	0.10 ± 0.70 *^2^	−1.25 ± 0.36
PC2	0.30 ± 0.89	−0.22 ± 1.17	−0.14 ± 0.86
PC3	0.36 ± 1.18	−0.26 ± 0.94	−0.16 ± 0.67
1.11 m·s^−1^			
PC1	0.88 ± 0.74 ^†^ *^1^	−0.09 ± 0.61 *^2^	−1.10 ± 0.42
PC2	0.28 ± 1.09 *^1^	0.16 ± 1.09 *^2^	−0.59 ± 0.60
PC3	0.35 ± 1.04	−0.33 ± 1.01	−0.05 ± 0.82
1.39 m·s^−1^			
PC1	0.82 ± 0.65 ^†^ *^1^	−0.01 ± 0.76 *^2^	−1.13 ± 0.38
PC2	0.37 ± 1.09 *^1^	0.05 ± 0.97	0.58 ± 0.65

^†^ Significant difference between SOG and MOG. *^1^ Significant difference between SOG and NWG. *^2^ Significant difference between MOG and NWG. MOG, moderately obese group; NWG, normal-weight group; PC, principal component (1, 2, 3); SOG, severely obese group.

## Abbreviations

α	Phase shift between E_k_ and E_p_ curves
BMI	Body mass index
COM	Center of mass
COM_l_	Center of mass lateral displacements
COM_v_	Center of mass vertical displacements
E_k_	Mechanical kinetic energy
E_kf_	Forward mechanical kinetic energy
E_kl_	Lateral mechanical kinetic energy
E_kv_	Vertical mechanical kinetic energy
E_p_	Mechanical potential energy
E_tot_	Total mechanical energy
F_f_	Forward ground reaction force
F_l_	Lateral ground reaction force
F_v_	Vertical ground reaction force
*g*	Gravity
GRF	Ground reaction force
*h*	Vertical position of the COM
iDXA	Dual-energy X-ray absorptiometry
KMO	Kaiser–Meyer–Olkin Test
LMM	Linear Mixed Model
*m*	Body mass
MET	Metabolic equivalent
MOG	Moderately obese group
NEAT	Non-exercise activity thermogenesis
NetC_w_	Absolute net energy cost of walking
NetC_w/kg_	Relative net energy cost of walking
NetE	Net locomotor efficiency
NWG	Normal-weight group
PC	Principal component
PC1	Principal component 1
PC2	Principal component 2
PC3	Principal component 3
PCA	Principal component analysis
RER	Respiratory exchange ratio
SD	Standard deviation
SMR	Absolute standing metabolic rate
SMR_/kg_	Relative standing metabolic rate
SOG	Severely obese group
TEE	Total energy expenditure
τ	Step period
t_pk+_	Difference between the time period at which E_k_ and E_p_ curves increase simultaneously
${\dot{V}\mathrm{CO}}_{2}$	Carbon dioxide production
${\dot{V}O}_{2}$	Oxygen uptake
${\dot{V}}_{E}$	Ventilation
V_f_	Forward velocity of the COM
V_l_	Lateral velocity of the COM
V_v_	Vertical velocity of the COM
W_ext_	Absolute external mechanical work
W_ext/kg_	Relative external mechanical work
W_k_	Absolute kinetic mechanical work
W_k/kg_	Relative kinetic mechanical work
W_p_	Absolute potential mechanical work
W_p/kg_	Relative kinetic mechanical work
W_tot_	Total mechanical work
